# Draft genome assemblies for two species of *Escallonia* (Escalloniales)

**DOI:** 10.1186/s12863-023-01186-7

**Published:** 2024-01-02

**Authors:** Andre S. Chanderbali, Christopher Dervinis, Ioana G. Anghel, Douglas E. Soltis, Pamela S. Soltis, Felipe Zapata

**Affiliations:** 1 Florida Museum of Natural History, University of Florida, Gainesville, FL USA; 2 School of Forest, Fisheries, and Geomatics Sciences, University of Florida, Gainesville, FL USA; 3 Department of Ecology and Evolutionary Biology, University of California, Los Angeles, CA USA; 4 Department of Ecology and Evolutionary Biology and Center for Tropical Research, Institute of the Environment and Sustainability, University of California, Los Angeles, CA USA

**Keywords:** Andes, Campanulidae, *Escallonia rubra*, *Escallonia herrerae*, Genomics, Oxford Nanopore Technology

## Abstract

**Objectives:**

*Escallonia* (Escalloniaceae) belongs to the Escalloniales, a diverse clade of flowering plants with unclear placement in the tree of life. *Escallonia* species show impressive morphological and ecological diversity and are widely distributed across three hotspots of biodiversity in the Neotropics. To shed light on the genomic substrate of this radiation and the phylogenetic placement of Escalloniales as well as to generate useful data for comparative evolutionary genomics across flowering plants, we produced and annotated draft genomes for two species of *Escallonia*.

**Data description:**

Genomic DNA from *E. rubra* and *E. herrerae* was sequenced with Oxford Nanopore sequencing chemistry, generating 3.4 and 12 million sequence reads with an average read length of 9.4 and 9.1 Kb (approximately 31 and 111 Gb of sequence data), respectively. In addition, we generated Illumina 100-bp paired-end short read data for *E. rubra* (approximately 75 Gb of sequence data). The *Escallonia rubra* genome was 566 Mb, with 3,233 contigs and an N50 of 285 Kb. The assembled genome for *E. herrerae* was 994 Mp, with 5,760 contigs and an N50 of 317 Kb. The genome sequences were annotated with 31,038 *(E. rubra*) and 47,905 (*E. herrerea*) protein-coding gene models supported by transcriptome/protein evidence and/or Pfam domain content. BUSCO assessments indicated completeness levels of approximately 98% for the genome assemblies and 88% for the genome annotations.

## Objective

Escalloniales comprise approximately 130 species of herbs, shrubs, and trees that grow in diverse habitats ranging from desolate rocky outcrops to rain forests across South America, Australia, Southeast Asia, and the Indian Ocean islands [1]. It is not known how and when Escalloniales diversified so extensively and colonized the Southern Hemisphere because the phylogenetic relationships within Escalloniales and between Escalloniales and other flowering plant lineages remain elusive. Escalloniales are part of the more inclusive clade Campanulidae, a hyperdiverse group of flowering plants with approximately 35,000 species [2]. Yet, the precise phylogenetic relationships among the major lineages of Campanulidae have not been clearly resolved with strong support by current molecular data [3–7]. Clarifying these relationships is critical to elucidate the mechanisms of phenotypic evolution and geographic diversification for a large group of angiosperms [8, 9]. Within Escalloniales, the genus *Escallonia* represents a remarkable radiation across three hotspots of biodiversity in the mountains of South America [10, 11]. *Escallonia* species grow from sea level to snow line, and from temperate to tropical regions, showing distinct adaptations related to environmental stress such as extreme temperature and water availability. Further, groups of closely related *Escallonia* species have diversified independently along elevational gradients in the tropical Andes, Southern Brazil, and the temperate Andes, suggesting that repeated ecological divergence may play an important role in *Escallonia* speciation [10]. Thus, *Escallonia* is emerging as a notable system to uncover the ecological and evolutionary processes underpinning tropical plant adaptation, speciation, and the nature of plant species [12]. To begin investigating the genomic substrate and biological processes underlying the radiations in *Escallonia* and Escalloniales, we hereby report the draft genomes of two Escallonia species. These data will also be relevant for broader comparative genomics studies across flowering plants.

## Data description

**Methodology** - Leaf tissues from a single *Escallonia rubra* plant and an *Escallonia herrerae* plant cultivated at the University of California Botanical Garden at Berkeley (Voucher numbers: UCBG92.1500 *E. rubra*, UCBG64.0493 *E. herrerae*) were used for genomic DNA extraction and sequencing (Table 1; Data File 1). For *E. rubr*a, isolated DNA was prepared following the Nextera XT DNA Library Prep Kit guideline and sequenced on an Illumina HiSeq 4000 system to generate 100-bp paired-end WGS reads (Table 1; Data Set 1; 376 million paired-end reads). In addition, we sequenced high-molecular-weight genomic DNA for both *E. rubra* and *E. herrerae* using the Oxford Nanopore Technology (ONT) PromethION 24 A series and the LSK114 ligation prep kit and R10.4.1 flow cells to generate approximately 140 Gb of sequence data (Table 1; Data Sets 2 and 3); 3.4 and 12 million sequence reads with an average read length of 9.4 and 9.1 Kb (approximately 31 and 111 Gb of sequence data), for *E. rubra* and *E. herrerae*, respectively. We used the Canu genome assembler [13] to generate contigs with ONT data. These were then polished (for *E. rubra*) using WGS sequences through NextPolish [14] and deduplicated using Purge Haplotigs [15].

### Genome descriptions

***Escallonia rubra*** – The *Escallonia rubra* genome assembly (Table 1, Data Set 4) consists of 3,233 contigs (N50 = 285 kb) with a total length of 566 Mb (Table 1, Data Set 5). The genome annotation includes 31,028 gene models supported by transcriptome and protein sequences and/or the presence of Pfam domains (Table 1; Data Set 6). BUSCO (Benchmarking Universal Single-Copy Orthologs) analyses based on conserved single-copy eudicot genes [16] indicate completeness levels of 97.8% for the genome sequence and 87.8% for the genome annotation (Table 1; Data Set 7).

***Escallonia herrerae*** - The *Escallonia herrerae* genome assembly (Table 1, Data Set 8) consists of 5,760 contigs (N50 = 317 kb) with a total length of 944 Mb (Table 1, Data Set 9). The genome annotation includes 47,905 gene models supported by transcriptome and protein sequences and/or the presence of Pfam domains (Table 1, Data Set 10). BUSCO analyses, relying on conserved single-copy eudicot genes [16], indicate completeness levels of 97.8% for the genome sequence and 87.8% for the genome annotation (Table 1, Data Set 11).

**Table 1:** Overview of data files/data sets

Label	Name of data file/data set	File types (file extension)	Data repository and identifier (DOI or accession number)
Data File 1	Materials and Methods	Text (.docx)	Figshare:10.6084/m9.figshare.24263800 [17]
Data set 1	Illumina reads of *E. rubra* genomic DNA	DNA sequence (.fastq)	NCBI:https://identifiers.org/ncbi/insdc.sra:SRX21711620 [18]
Data set 2	ONT reads of *E. rubra* genomic DNA	DNA sequence (.fastq)	NCBI:https://identifiers.org/ncbi/insdc.sra:SRX21711620 [19]
Data set 3	ONT reads of *E. herrerae* genomic DNA	DNA sequence (.fastq)	NCBI:https://identifiers.org/ncbi/insdc.sra:SRX21711620 [20]
Data set 4	*E. rubra* whole genome sequence	DNA sequence (.fasta)	NCBI:https://identifiers.org/ncbi/insdc.gca:GCA_033065855.1 [21]
Data set 5	Assembly metrics for *E. rubra* whole genome sequence	Spreadsheet (xlsx)	Figshare:(10.6084/m9.figshare.24263788.v1) [22]
Data set 6	Annotation of *E. rubra* whole genome sequence	General Feature Format (.gff)	NCBI:https://identifiers.org/ncbi/insdc.gca:GCA_033065855.1 [21]
Data set 7	BUSCO summary statistics for *E. rubra* whole genome sequence and annotated proteins	Portable Network Graphic (.PNG)	Figshare:(10.6084/m9.figshare.24265801.v1) [23]
Data set 8	*E. herrerae* whole genome sequence	DNA sequence (.fasta)	NCBI:https://identifiers.org/ncbi/insdc.gca:GCA_033070095.1 [24]
Data set 9	Assembly metrics for *E. herrerae* whole genome sequence	Spreadsheet (xlsx)	Figshare:(10.6084/m9.figshare.24263785.v1 [25]
Data set 10	Annotation of *E. herrerae* whole genome sequence	General Feature Format (.gff)	NCBI:https://identifiers.org/ncbi/insdc.gca:GCA_033070095.1 [24]
Data set 11	BUSCO summary statistics for *E. herrerae* whole genome sequence and annotated proteins	Portable Network Graphic (.PNG)	Figshare:(10.6084/m9.figshare.24265804.v1) [26]

## Limitations

The base chromosome number of *Escallonia* is *n* = 12 [27], but our assemblies consist of 3,233 and 5,760 contigs for *E. rubra* and *E. herrerae*, respectively. As such, additional genome assembly and sequencing technologies, such as Hi-C, are needed to generate chromosome-level assemblies suitable for chromosome-scale comparative genomics.

## Data Availability

The data described in this Data Note can be freely and openly accessed at NCBI under accession number PRJNA1014744. Please see Table 1 and references [17–25] for details and links to the data. Detailed methodology is available on the Figshare repository [17].

## Abbreviations

ONT: Oxford Nanopore Technology
BUSCO: Benchmarking Universal Single-Copy Orthologs
WGS: Whole Genome Shotgun
